# Bright’s Disease of the Kidneys

**Published:** 1886-03

**Authors:** 


### BRIGHT'S DISEASE OF THE KIDNEYS.

Dr. J. A. Regan, A.M., M.D., of Weaverville, North Carolina, writes as follows in the *Medical Summary*, of Bright's Disease of the Kidneys:

"I wrote an article in the *Medical World*, or rather that was published in it in the early part of 1874, calling attention to *Malaria* as a cause of Bright's Disease of the Kidneys, and asking the experience of those physicians who lived in non-malarial sections, but to the present I have had no responses. I was led to investigate this from the fact that I have been practicing medicine for over thirty years in Western North Carolina, a non-malarial section, and in that time have had only two cases of Bright's disease, and both of them were from malarial sections, neither had been but a short time in this country. Since the publication of that article, I have had a conversation with Dr. Anderson, who was raised here and has practiced medicine for twenty-five years, and he tells me he never saw a case of Bright's disease in his practice. Dr. Cain, formerly of Charleston, South Carolina, who has been practicing here for twenty-one years, says he has only had three cases, and they had all been exposed to malarial influences. Dr. Weaver, who has been practicing here for ten years, says he has never seen a single case. I have talked with some twenty physicians in this section, and none have ever seen a single case that has not been exposed to malarial influences. Now taking all the facts, may not malaria be one of the prime causes of Bright's disease? I do dot say it is the only cause, but I do think it one of the leading causes of this frightful disease; and its scarcity in this non-malarial section confirms me in my opinion. I find no author who has taken this view of the subject except some German professor, whose name I do not now remember; and his article appeared some six months after my former article was published. He stated that he had been investigating the disease for several years, and his conclusions were that it was of malarial origin. If malaria proves to be the cause of Bright's disease of the kidneys, will it not entirely change the treatment? And may we not expect better success in treating it? Dr. W. W. Wing, formerly of Norfolk, Va., since I called his attention to it, some two years ago, tells me that the more he studies the subject the more thoroughly he is convinced that it is a disease of malarial origin. It is known that malaria may be dormant in the human system for years, and then develop itself in some way. All of us have witnessed such cases. Let it be thoroughly investigated."

